# The Impact of Various Types of Cooking on the Fate of Hg and Se in Predatory Fish Species

**DOI:** 10.3390/foods13030374

**Published:** 2024-01-24

**Authors:** Mariana Ribeiro, Laurène Douis, José Armando Luísa da Silva, Isabel Castanheira, Axelle Leufroy, Petru Jitaru

**Affiliations:** 1 Laboratory for Food Safety, University Paris Est Creteil, Anses, 94700 Maisons-Alfort, France; mendesribeiro@tecnico.ulisboa.pt (M.R.); laurenedouis94@gmail.com (L.D.); axelle.leufroy@anses.fr (A.L.); 2 Department of Food and Nutrition, National Institute of Health Doutor Ricardo Jorge, INSA IP, Av. Padre Cruz, 1649-016 Lisbon, Portugal; isabel.castanheira@insa.min-saude.pt; 3 Centre for Structural Chemistry, Institute of Molecular Sciences, Chemical Engineering Department, Higher Technical Institute, University of Lisbon, Av. Rovisco Pais, 1049-001 Lisbon, Portugal; pcd1950@tecnico.ulisboa.pt

**Keywords:** swordfish, tuna, dogfish, ICP-MS, food composition, culinary treatments

## Abstract

This study addresses the effect of various cooking approaches on total Hg (Hg_T_) and total Se (Se_T_) contents in three predatory fish species. For this purpose, samples of swordfish, dogfish, and tuna from regular French (fish) markets were cooked by boiling, steaming, grilling, and frying, respectively. The levels of Hg_T_ and Se_T_ in raw and cooked samples were determined by inductively coupled plasma-mass spectrometry. The data showed a significant increase in Hg_T_ and Se_T_ levels between raw and cooked samples (33% of the samples for Se_T_ and 67% for Hg_T_) due to the water loss during the cooking. High intra-species variation related to Hg_T_ and Se_T_ levels was found. Considering the level of exposure to Hg_T_ through fish consumption and taking also into account the possible protective effect of Se (expressed here via the Se/Hg molar ratio), the safest cooking approach corresponds to grilled swordfish, fried tuna, and steamed dogfish, which show Se/Hg molar ratios of (1.0 ± 0.5), (4.3 ± 4.2), and (1.0 ± 0.6), respectively.

## 1. Introduction

Seafood is an important part of people’s diets, with a continued increase over the years in its consumption amount per capita. The current worldwide average fish consumption is estimated at ≅21 kg wet weight (ww) per person/year and is expected to continue to rise [1]. Fishery products are a source of nutrients, such as proteins, lipids, vitamins, and numerous micronutrients including selenium, phosphorus, potassium, sodium, calcium, magnesium, iron, and iodine, making it a food of high nutritional value. However, several chemical pollutants, which are continuously being introduced into marine ecosystems both by natural and anthropogenic sources, can be accumulated by marine species through a variety of pathways and hence biomagnified in the food chains. Highest concentrations are generally found at the top of aquatic food webs, making the consumption of predatory fish (e.g., shark, swordfish, and tuna) possibly hazardous for human health [2,3,4,5,6].

Special attention is focused on mercury (Hg), which is a toxic trace metal that presents the highest concern to human health with regards to seafood consumption. An increase of Hg levels in several fish species has been observed around the world in the last decades, some exceeding the threshold of adequacy for human consumption (0.5 to 1.0 mg/kg ww, depending on the fish species) established by the World Health Organization (WHO) and legislated by the European Commission regulation EC/1881/2006 [3,5,7,8,9,10,11].

In the aquatic environment, inorganic Hg (Hg^2+^) can be readily transformed into methylmercury (MeHg), which is the most toxic Hg species and which is also mostly biomagnified by fish [5,8,9,10,12]. Studies have found that selenium (Se), also present in predatory fish, may have the ability to decrease or prevent the toxic effects of Hg [13]. The protective effects of Se species against Hg toxicity have been attributed to various factors, such as the competition for binding sites, the reduction of oxidative stress, or the formation of Hg-Se complexes, which are metabolically inert [14,15]. An alternative theory suggests that an excess of bioavailable Se could compensate the lack of Se taken up by Hg, ensuring selenoproteins synthesis and hence the normal antioxidant functions of selenoenzymes. Some authors suggested that the protective effect of bioavailable Se against Hg is effective when the Se and Hg levels in target tissues exceed the stoichiometry of 1:1 [14,15,16,17,18,19].

Depending on dietary habits, fish can be consumed in a raw state (e.g., in sushi meal) or cooked in various ways such as by boiling, grilling, steaming, baking, or frying. The cooking mode can lead to variations in Hg concentrations including its speciation. In addition, different compositions in fish Se content can also affect the fate of Hg during culinary treatments [2,3,5].

Even though the Hg toxicity varies as per its different chemical forms, the majority of studies regarding its impact via fish ingestion have been related to total Hg (Hg_T_) levels in raw fish [2]. Additionally, the previous studies do not account for the Se presence even if Hg toxicity was found to be reduced by this element naturally present in fish [2,3,5]. Furthermore, there are still many controversies about the influence of fish cooking on the levels of Hg and Se. While some studies reported lower levels of Hg and Se in cooked fish compared to the raw material, others reported their increase [2,20,21]. Several variables (temperature, method of cooking, time, seasoning, etc.) in culinary processing can influence the levels of these elements in cooked fish [22]. Therefore, it is important to better understand the mechanisms related to Hg toxicity in cooked fish and particularly the antagonism between Hg and Se in marine predatory fish species, which are known to significantly bioaccumulate MeHg. 

This study aims to assess the impact of various cooking methods such as boiling, steaming, and grilling on the fate of Hg and Se in different species of predatory fish. The impact of cooking was assessed in two ways: firstly, by determining the Hg_T_ and Se_T_ levels in fish cooked as it would be consumed (food composition data) and comparing it with the raw fish, and secondly, by evaluating the outcome of the net gain or loss in terms of Hg_T_ and Se_T_. The influence of Se on the fate of Hg during the cooking was also addressed to study the potential protective effect of Se against Hg toxicity. The results provide useful information on health risks associated with human exposure to Hg via the consumption of cooked fish, which may enable food authorities to better advise the general population regarding the consumption of predatory fish species.

## 2. Materials and Methods

### 2.1. Instrumentation

An ICP-MS (7700x model from Agilent Technologies, Courtaboeuf, France) equipped with a third-generation Octopole Reaction System (ORS3) was used for the determination of Hg_T_ and Se_T_. The ICP-MS was provided with an integrated Sample Introduction System (ISIS) to reduce the analysis time. 

Samples digestion was carried out using a Multiwave Pro (Anton Paar, Courtaboeuf, France) equipped with 80 mL quartz vessels (80-bar operating pressure). 

The optimum analytical conditions for the ICP-MS/MS method are provided in Table 1.

### 2.2. Chemicals and Reagents

All solutions were prepared using analytical reagent grade chemicals and ultrapure water (18.2 MΩ cm^−1^) prepared by Milli-Q™ Integral 5 Elix Technology (Merck Millipore, Saint Quentin in Yvelines, France). Nitric acid (HNO_3_) (67% *v*/*v*, suprapur) was purchased from VWR (Fontenay-sous-Bois, France). The ICP-MS ultrapure grade (99.9995%) argon (Ar) and helium (He) were supplied by Linde (Montereau, France).

Intermediate standard solutions for calibration (Hg and Se) and internal standardization containing scandium (Sc) and bismuth (Bi) were prepared using 1000 mg/L individual stock solutions purchased from LGC Standards (Molsheim, France). Calibration standard solutions were prepared daily in 6% (*v*/*v*) HNO_3_.

Certified reference materials (CRMs) ERM^®^-BB-422 (fish muscle) from the European Joint Research Centre (JRC), 7402-a (codfish tissue) from the National Metrology Institute of Japan, and DOLT-5 (Dogfish liver tissue) and DORM-4 (fish protein) from the National Research Council Canada were all purchased from LGC Standards (Molsheim, France).

### 2.3. Sampling 

Three predatory fish species were selected due to their high levels of Hg [2], but also different levels of Se and lipids, in order to investigate a possible influence of these factors in the behavior of Hg throughout the cooking process: swordfish (*Xiphias gladius*, Linnaeus, 1758), dogfish (*Scyliorhinus canicular*, Linnaeus, 1758) and tuna yellowfin (*Thunnus albacares*, Bonnaterre, 1788).

All fish were purchased from various markets from Maisons-Alfort (Parisian area) between April and May 2021. Based on the vendors’ information, the swordfish were caught in the Pacific and Indian Oceans, tuna were caught in the Indian Ocean, while dogfish originated from France, except for one sample that was caught in The Netherlands.

Five different (or parts of) individuals per fish species (calculating a total of 15 fish) were purchased and studied further in order to obtain representative results.

### 2.4. Analytical Procedures

#### 2.4.1. Sample Preparation

The dogfish was bought dressed (i.e., viscera, scales, head, tail and fins removed), and its backbone was taken away. Swordfish and tuna were acquired as fillets (boneless sides of fish, without skin). All fish samples were separated into 4 parts (cross section of fillets): one was kept raw, while the others were used for cooking by boiling, grilling, steaming, and frying [23]. Furthermore, the fish sticks to be cooked were divided into two pieces of ≅50 g in order to obtain two (cooking) replicates. 

It is important to mention that the fish samples used for frying are different (purchased later on) from those used in the other cooking methods (boiling, grilling, and steaming) because the fish initially purchased was not sufficient to perform all the cooking treatments proposed in the study.

#### 2.4.2. Culinary Treatments

Fish were subjected to the four common culinary treatments described below (each cooking was carried out in duplicate):

(i) Boiling: 300 g of ultra-pure water was placed in a 500 mL beaker and brought to boiling. Then, the sample was immersed into the boiling water during 15 min. The internal temperature of the fish fillets was recorded at the beginning and every 5 min.
(ii) Frying: 300 mL of sunflower oil was placed into a 500 mL beaker and brought to a temperature of 130 °C. Then, the sample was immersed during 5 min; the internal temperature of the fish fillets was recorded at the start, halftime, and at the end of the cooking procedure.
(iii) Grilling: Each sample was cooked without any addition of sunflower oil or other ingredient for 15 min in a conventional (Teflon) frying pan. They were first cooked for 5 min each side, and then 2.5 min each side, for a total of 15 min. This allowed a homogenous cooking throughout the sample and prevented it from burning. The internal temperature of the fish fillets was measured each time the sample was turned.
(iv) Steaming: The steam cooker was filled with ultra-pure water up to its minimum level. The apparatus was heated until steam was produced (≅3 min), and then the sample was introduced into the adequate plastic recipients and cooked for 15 min; the temperature was checked every 5 min during the sample steaming. The sample was hold on a metal-free (plastic) holder to avoid any contamination with trace metals, including Hg.

The temperatures were recorded for all procedures during the culinary treatments. The maximum internal measured temperatures were 101 °C, 88 °C, 100 °C and 103 °C for boiling, grilling, steaming and frying process, respectively.

#### 2.4.3. Determination of Hg_T_ and Se_T_ by ICP-MS 

ICP-MS measurements of Hg_T_ and Se_T_ were performed using an accredited method (French accreditation committee, COFRAC) described previously [24]. Briefly, 0.3 g of raw or cooked sample previously freeze-dried were digested with HNO_3_ 67% (m/m) in a closed microwave system. The internal standards (IS) were added and then the digested sample were diluted to 50 mL with ultrapure water. 

The levels of Hg_T_ and Se_T_ were also determined in the ultra-pure water used for the boiling and steaming of the fish sample and also in the oil employed for the boiling. They were systematically lower than the method limit of quantification and hence not reported in this study.

A solution of Au at 20 µg/mL prepared in 10% (*v*/*v*) HNO_3_ was used throughout to rinse the system between each measurement to reduce memory effects [25,26]. The ICP-MS “Intelligent Rinse” function was also used allowing the rinsing step to continue between each measurement as long as the signal of Hg had not fallen below a certain threshold.

#### 2.4.4. Internal Quality Control

The laboratory implemented quality assurance and procedures framed on ISO 17025:2017 requirements, guaranteeing that values comply with international guidelines (WHO databases, EFSA or EuroFIR guidelines). Furthermore, short-term stability tests of the instrument were performed daily using a tuning solution to optimize the equipment, maximizing ion signals and to minimizing interference effects. The acceptance criteria for each equipment fit less than 10% of the dispersion coefficient of target uncertainty.

Additionally, several internal quality controls (IQCs) were established. Each analysis run included blank solutions to monitor the eventual cross-contamination or memory effects, certified reference materials (CRMs) and spiked samples to guarantee method trueness, duplicate samples to check the repeatability, and, also, the analysis of a standard containing Hg and Se (at 5.0 μg/L) every 8 to 10 samples in order to detect a possible instrumental signal drift.

The analyses were carried out by ICP-MS using the external calibration approach in the range 1.0–10 μg/L for both Hg_T_ and Se_T_ (r^2^ ≥ 0.995).

### 2.5. Statistical Data Treatment

Statgraphics Centurion^®^ software 19 (Statgraphics Technologies, Inc., The Plains, VA, USA) was utilized for the statistical analysis. An initial assessment of normality for all data sets was performed using the Shapiro-Wilk test. 

Paired *t*-test (data normal distributed) or paired-Wilcoxon test (data not normal distributed) were carried out in order to observe any impact of the culinary treatments in the three fish species. A 95% confidence interval was used for all analyses, and a significance level of *p* < 0.05 was considered as significant.

## 3. Results and Discussion

### 3.1. Influence of Various Cooking Modes on Hg_T_ and Se_T_ Levels

**Figure 1:**
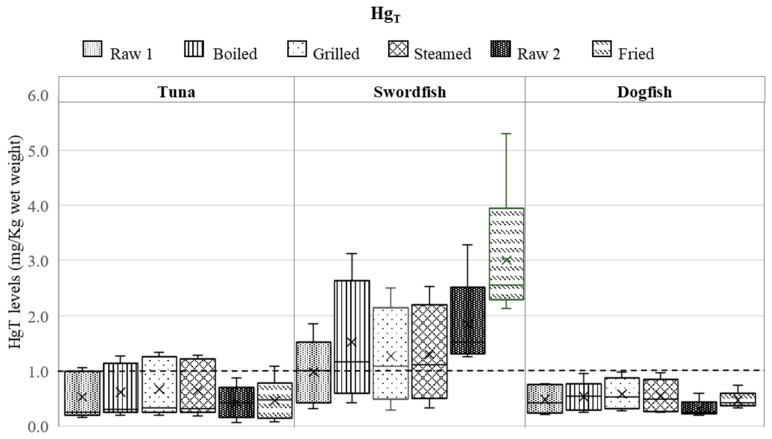
Levels of Hg_T_ (mg/kg ww) measured in raw (Raw 1 and Raw 2, *n* = 5) and cooked fish samples (*n* = 5, in duplicate). The x in the box plot represents the mean, and the solid black line represents the median. The black dashed line indicates the maximum levels for Hg established by Regulation EC/1881/2006 (1.0 mg/kg ww).

**Figure 2:**
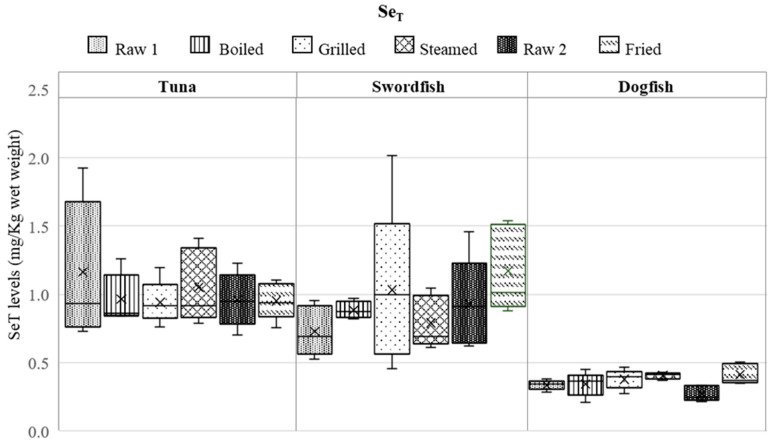
Levels of Se_T_ (mg/kg ww) measured in raw (Raw 1 and Raw 2, *n* = 5) and cooked fish samples (*n* = 5, in duplicate). The x in the box plot represents the mean, and the solid black line represents the median.

The levels of Hg_T_ and Se_T_ measured in raw and cooked fish samples are reported in Figure 1 and Figure 2, respectively.

The data related to raw samples are expressed with the measurement associated uncertainty (*n* = 5), while the results related to the cooked samples are the mean of two cooking replicates (*n* = 2). As was mentioned earlier, for the frying, the (5) samples of different fish individuals were purchased approximately one month later because of the insufficient sample amount purchased initially to carry out all the culinary treatments. The two batches of raw fish samples will be addressed further as Raw 1 and Raw 2.

The Hg_T_ levels in raw swordfish individuals varied between 0.31 ± 0.06 and 3.3 ± 0.7 mg/kg; for tuna the Hg_T_ ranged from 0.062 ± 0.013 to 1.1 ± 0.2 mg/kg, while for dogfish it ranged from 0.20 ± 0.04 to 0.76 ± 0.15 mg/kg. 

From the three fish species, the highest Hg_T_ levels in the raw samples were found in swordfish (mean concentration = 1.4 ± 0.8 mg/kg, *n* = 10) followed by tuna and dogfish, and this distribution agrees well with the previously published data [27]. Despite the high intra-species variation of the Hg_T_ levels in raw fish (between 56% and 74%), Hg_T_ levels measured in 80% of the swordfish raw samples were above the EU regulated maximum level (1.0 mg/kg). 

The mean values (*n* = 10) of Hg_T_ levels measured in raw tuna and dogfish were 0.48 ± 0.35 mg/kg and 0.40 ± 0.23 mg/kg, respectively. In addition to the swordfish and one tuna fish individual, the Hg_T_ levels in the other fish samples were below the maximum regulated level. Thus, only for the dogfish species, Hg_T_ in all five individuals were in agreement with the EU legislation.

Regarding the Se_T_ contents in the raw samples, they ranged from 0.526 ± 0.158 to 1.46 ± 0.44 mg/kg in swordfish, from 0.70 ± 0.21 to 1.9 ± 0.6 mg/kg in tuna, and from 0.22 ± 0.07 to 0.38 ± 0.11 mg/kg in dogfish. The Se_T_ mean values were 0.83 ± 0.28 mg/kg, 1.1 ± 0.4 mg/kg, and 0.30 ± 0.06 mg/kg in swordfish, tuna, and dogfish, respectively. Tuna is the fish species containing the highest Se_T_ level, followed by swordfish, whereas dogfish showed the lowest amount of Se_T_, as it was also reported by ANSES and EFSA (tuna (0.80 mg/kg) < swordfish (0.53 mg/kg) < dogfish (0.29 mg/kg)) [28,29]. 

As can be seen from Figure 1 and Figure 2, both Hg_T_ and Se_T_ levels in raw fish show high intra-species variability and also significant variation among the three fish species. Yet, as observed in other studies, the Se_T_ levels found in fish varied less than the Hg_T_ levels amongst the same fish species [30]. It is important to note that although fish were collected from nearby fish markets, the presence of Se and Hg elements in fish can be influenced by various factors. The levels of Hg and Se may depend on the seasonality because fish’s migratory patterns (and food habits) vary across seasons, leading to fluctuating levels of Se and Hg [15,30,31,32,33,34]. It is very likely that storage conditions from fishing to the fish market have no impact on the levels of Hg and Se in fish, taking into account that these elements are strongly bound to the proteins.

With respect to the cooked samples, Figure 1 reveals high data dispersion in all Hg_T_ concentrations with RSDs ranging from 33% in fried dogfish to 84% in fried tuna. The data bar for errors was larger in fried swordfish, indicating a wide dispersion of data points around the mean value. This suggests that the data are variable and not tightly clustered. Although less expressive, as can be observed in Figure 2, high data dispersion was also found for Se_T_ in most cases, with the exception of boiled swordfish and steamed dogfish (RSD < 10%). 

Good repeatability related to Se_T_ and Hg_T_ levels was observed between replicates in all culinary treatments. This indicates that repeatable cooking methods were implemented. Dogfish replicates showed RSD < 10% (*n* = 5) in all culinary treatments. For swordfish, slight heterogeneity in Se_T_ replicates was observed for grilling and frying (RSD = 13% and 16%, respectively; *n* = 5). Regarding tuna, the replicates variation of Se_T_ was >10% for steaming and frying (RSD = 14% and 15%, respectively; *n* = 5) and for Hg_T_ with frying (RSD = 14%; *n* = 5). 

With regards to the food composition data on Hg_T_ levels in cooked fish, significantly higher concentrations were measured in cooked tuna by boiling and steaming, while grilling and frying led to significantly higher levels in cooked dogfish (*p* < 0.05). In case of swordfish, all cooking modes increased significantly the Hg_T_ levels compared to the raw samples (*p* < 0.05). Actually, out of 12 sample types (tuna, swordfish, and dogfish cooked by boiling, grilling, steaming, and frying), only in four cases no significant differences were observed between raw and cooked samples, namely, two tuna cooked samples (grilled and fried) and two dogfish cooked samples (boiled and steamed), while for the rest, an increase in Hg_T_ levels was observed after cooking. 

The levels of Se_T_ in swordfish were significantly higher in the fried samples (*p* < 0.05), compared to the raw ones. In dogfish, it was found that the Se_T_ concentrations of grilled, steamed, and fried samples are significantly higher than the respectively raw samples. However, in tuna samples there were no significant differences in Se_T_ levels before and after cooking (*p* ≥ 0.05). From the 12 cases examined, 8 cases did not show any substantial variations. Thus, only four cases showed significant increases of Se_T_, namely, one fried swordfish sample and three dogfish cooked samples (grilled, steamed, and fried). 

It can also be observed from Figure 1 and Figure 2 that regardless of the culinary treatment applied, its influence on the concentrations of Hg_T_ or Se_T_ differs from matrix to matrix, and vice-versa, the same matrix behaves differently, depending on the culinary treatment. Furthermore, no correlation was observed between the behavior of Hg_T_ and Se_T_ during the cooking process, which means that the two elements exhibit different behaviors when subjected to the same culinary treatment. Also, it is important to note that when assessing the impact of fish cooking on the fate of Hg and Se, the intra-species variability must also be taken into consideration.

### 3.2. Assessment of Se/Hg Molar Ratios in Raw and Cooked Fish

Several authors reported that the protection provided by Se against Hg toxicity depends upon the concentration ratio between the two elements and needs to exceed a stoichiometry (molar ratio) of 1:1 to be effective [19,30]. It is worth to note that several factors (such as fish size, age, gender, habitat, and migration pattern) make it unlikely to obtain fish of the same species with the same range of Hg and Se levels. As already highlighted, there are intra-species and inter-species variability, and the molar ratios are not homogenous within the same fish species and are even less consistent among different fish species [30]. The Se/Hg molar ratios related to the cooking of the fish species in our study are presented in Table 2.

The highest Se/Hg molar ratios were measured in tuna fish samples, being always considerably >1, suggesting that Se is always in excess relatively to Hg in this species. However, a significant decrease is observed in boiled, grilled, and steamed tuna samples, when comparing to the raw ones (*p* < 0.05). 

In swordfish, a Se/Hg molar ratio ≥1 is found solely in the Raw 1 sample, while in the Raw 2 sample, the Se/Hg ratio was significantly lower (0.55). In this fish species, only frying led to a significant decrease in Hg/Se molar ratio (*p* < 0.05), while other cooking modes had no significant impact. 

Regarding dogfish, in both raw samples, the Se/Hg molar ratio was ≥1; only boiling and grilling decreased significantly the Se/Hg molar ratio (*p* < 0.05) compared to the corresponding raw samples.

Some authors reported that fish with high Hg levels exhibit a lower Se/Hg molar ratio compared to fish with generally low Hg levels [30], which is consistent with our results. Actually, from the three fish species analyzed, swordfish exhibited the lowest Se/Hg molar ratio, and it was the fish with the highest Hg levels. In addition, previous investigations concluded that fish species positioned higher in the food chain typically have lower molar ratios. Swordfish and tuna share the same level in the food chain, while, dogfish occupy a lower position [30,35]. In our study this was not observed because tuna fish showed the highest Se/Hg molar ratios.

From the composition data (Figure 1 and Figure 2), it can be seen that Hg_T_ levels in swordfish increased in the cooked samples (compared to the raw samples) regardless of the culinary treatment, whereas Se_T_ increased only in the fried samples, indicating that this cooking mode (frying) is the best choice for this fish species in terms of protective effect of Se against Hg. However, as can be seen in Table 1, the Se/Hg molar ratio in the fried sample of swordfish is low (0.4 ± 0.1) and moreover, it decreased compared to the raw sample. Thus, the use of Hg_T_ and Se_T_ levels to describe the antagonism of these elements is not sufficient, the molar ratio being a better indicator for this purpose. In this context, grilled swordfish consumption can be considered as the safer choice.

For tuna fish, the most optimum cooking mode is frying, taking into account that for this fish species, Hg_T_ decreased as a result of these treatments while the Se_T_ levels remained constant. At the same time, a significant decrease (*p* < 0.05) in the Se/Hg molar ratio was observed in boiled, grilled, and steamed tuna samples, when comparing to the raw ones, hence confirming that in this case frying is the optimal mode. Other studies in different fish species reported also that frying results in a decrease in the concentration of Hg [36]. In our study, out of the four culinary treatments tested, frying achieved the highest internal temperature. Studies have shown that heating proteins can change the strength of their interaction with Hg, hence resulting in increased solubilization of Hg and a subsequent decrease in Hg levels in fried fish. In addition to protein content, the higher fat content in fried fish compared to raw or other cooked fish may also affect the solubility of Hg [5,37]. The observed decrease in Hg_T_ in fried tuna could be explained by these factors (combined or individually). However, in our study, we only observed these findings in tuna fish. Finally, regarding the dogfish, boiling and steaming did not lead to any increase in Hg_T_ level, compared to grilling and frying, which led to a slight increase in Hg_T_ levels. Yet, the Se/Hg molar ratio was ≥1 only in the steamed dogfish, hence indicating that dogfish steaming could be the safest cooking mode. 

The behavior of Hg during cooking is influenced by the water, protein, and lipid contents of fish [5]. Thus, variations in the response to different culinary treatments among fish species can be explained by differences in macronutrient composition [28]. In addition to considering Se/Hg molar ratios, a comprehensive benefit-risk evaluation should be conducted to define the Acceptable/Tolerable Daily Intake (ADI/TDI) and risk characterization parameters of Hg in cooked fish, taking into account the beneficial features of Se presence in fish. 

### 3.3. Assessment of the Net Gain or Loss in Terms of Hg_T_ and Se_T_ in the Cooked Fish Samples

Most of the studies assessing the levels of Hg in fish after undergoing various cooking methods reported differing findings; while in some studies a lower level of Hg was found in cooked fish, others reported its increase or no significant changes [2].

Our results showed that in all cases where significant differences between raw and cooked samples were found, they were due to an increase in Hg_T_ or Se_T_ concentrations after the culinary treatment, as observed also by other authors [5,20,21,30]. Actually, in most cases the sample weight in cooked samples is lower than in raw samples due to drying, hence leading to higher Hg concentrations in the cooked samples. As a consequence, it is difficult to directly compare raw and cooked samples in terms of Hg_T_ or Se_T_ levels because they have different moisture, fat, or chemical composition [38]. Another potential explanation for this outcome could be that cooking causes the loss of minerals (Na, K, Mg, P, etc.) from the fish matrix resulting also in a pre-concentration effect of Hg in the cooked sample without actual increase of the Hg (absolute) amount in the cooked fish [21]. 

Taking into account that in most cases the fish is consumed cooked, it is important to understand the actual impact of the culinary treatments on the Hg_T_ or Se_T_ levels in fish. For this purpose, we calculated the cooking factors (CF, %) reflecting the relative level of these elements in the cooked samples compared to the raw samples (Equation (1), [38]).

CF (%) = m_cooked_/m_raw_ × 100 (1)

where

m_cooked_, mass of cooked sample (g);

m_raw_, mass of raw sample (g).

The levels of Hg_T_ and Se_T_ obtained in the cooked samples were multiplied by the corresponding cooking factor (Equation (1)) to allow a direct comparison between raw and cooked fish. Additionally, to easily evaluate the net gain or loss or cooking balance (CB, %) related to Hg_T_ or Se_T_ during the various culinary treatments, the following equation (Equation (2), [38]) was applied:

CB (%) = (C_f_/C_i_) × 100 (2)

where:

C_f_, level of Hg_T_ and Se_T_ (mg/kg) in the cooked sample;

C_i_, level of Hg_T_ and Se_T_ (mg/kg) in the raw sample.

**Figure 3:**
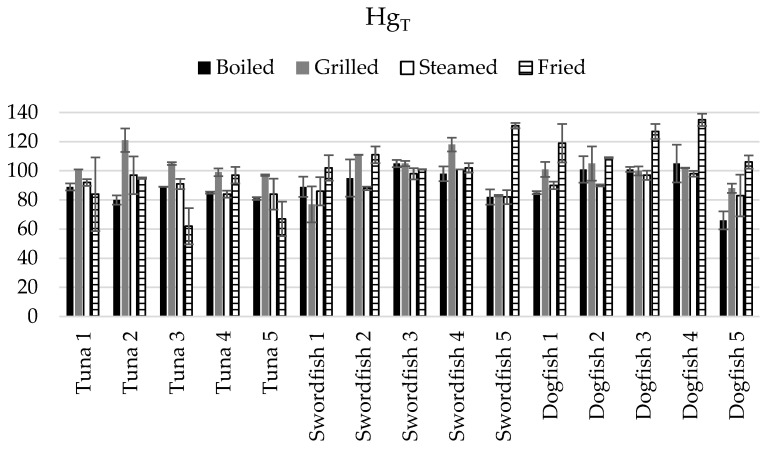
Cooking balance (CB, %) related to Hg_T_ levels in cooked samples of tuna, swordfish, and dogfish by boiling, grilling, steaming, and frying (the error bars represent the standard deviation of the cooking replicate, *n* = 2).

Values of CB < 100% indicate a net reduction in Hg_T_ or Se_T_ levels in fish as a result of the sample’s cooking, while CB > 100% indicates a pre-concentration of Hg_T_ or Se_T_ in the cooked matrix (CB = 100% indicates no cooking impact). Figure 3 reports the cooking balance data related to Hg_T_ levels in cooked samples of tuna, swordfish, and dogfish by boiling, grilling, steaming, and frying.

Regarding the impact of cooking on the net Hg_T_ levels (Figure 3), a high intra-species variability was noticed for fried tuna, grilled swordfish, and boiled dogfish. In these three groups of samples, some fish individuals showed the highest Hg_T_ loss due to cooking. The Hg_T_ intra-species losses or gains outcome after cooking varied between −38% and −3% in fried tuna, from −23% to 18% in grilled swordfish, and finally, in a range of −24% to 5% in boiled dogfish. 

The observed losses of Hg during cooking may be due to its volatile nature, as high temperatures can cause it to evaporate.

**Figure 4:**
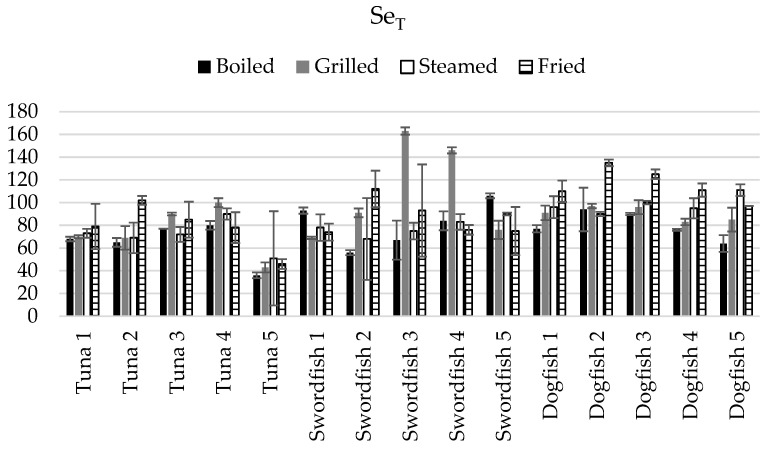
Cooking balance (CB, %) related to Se_T_ levels in cooked samples of tuna, swordfish, and dogfish by boiling, grilling, steaming, and frying (the error bars represent the standard deviation of the cooking replicates, *n* = 2).

Figure 4 presents the cooking balance related to Se_T_ levels in cooked samples of tuna, swordfish, and dogfish by boiling, grilling, steaming, and frying.

High intra-species variability regarding Se_T_ levels was also observed in all culinary treatments performed in tuna (in all cases, RSD = 20–30%) and in swordfish (RSD = 20–40%) with the exception of steaming (RSD = 10%). On the opposite, all the culinary treatments performed in dogfish showed relatively good intra-species homogeneity (RSD < 15%). The grilled swordfish showed the highest intra-species variability, with gains and losses of Se_T_ ranging from −31% to 63%. Thus, contrary to what was observed when assessing the Hg_T_ and Se_T_ levels solely, the impact of cooking on the net Hg_T_ is intra-species more uniform (9/12 cases) when compared to Se_T_ (5/12). However, as observed in the case of Hg_T_ and Se_T_ levels in cooked fish, inter-fish species and inter-culinary treatment differences were also found to be significant.

In terms of the average Hg_T_ loss or gain outcome after cooking, it varied between–19% (fried tuna) to 19% (fried dogfish). A significant decrease in average Hg_T_ levels after cooking was also observed in boiled tuna (−15%). On the other hand, as well as in the case of fried dogfish, no decrease in Hg_T_ was observed in grilled tuna and fried swordfish.

Although in most cases no major loss of Hg_T_ has been observed during cooking, the Hg present in fish can undergo “intra-species conversion” during cooking, converting Hg from its organic form (MeHg), which is more readily absorbed and toxic, to its inorganic form, which is less toxic.

The highest Se_T_ loss was found in the cooked tuna samples, particularly the fifth tuna individual, which had a significant loss (49 to 64%) of Se_T_ in all culinary treatments. On average, the highest Se_T_ amount lost during cooking occurred in the boiled tuna samples (−35%). As was observed for Hg_T_, no significant Se_T_ losses were found in fried dogfish. 

It is worth mentioning that, when comparing the effects of cooking taking into account the net Hg_T_ and Se_T_ levels, significantly (*p* < 0.05) higher losses of Se_T_ due to cooking were globally observed, except for the cases of fried tuna, grilled swordfish, and steamed and fried dogfish.

The net outcome related to Hg_T_ and Se_T_ gain and loss reinforce the previous recommendations of safest fish consumption choices based on the molar ratios. For instance, the most important loss of Hg_T_ and the least loss of Se_T_ were found in fried tuna samples, which may indicate a detoxification due to this cooking mode. The Se/Hg molar ratio in the fried tuna (4.3 ± 4.2) was not significantly different (*p* ≥ 0.05) from the Se/Hg molar ratio in the raw samples (4.7 ± 5.3). 

In swordfish, our results show Se_T_ loss due to cooking but not Hg_T_, hence indicating no impact of cooking on lowering of the Hg toxicity for this fish species. This is consistent with the molar ratios data (Table 1), where a decrease was observed, in some cases significant (*p* < 0.05), in the molar ratios in the swordfish cooked samples compared to the raw swordfish. 

Finally, from the four culinary treatments employed in this study, boiled and steamed dogfish showed the highest net Hg_T_ loss, but contrary to boiled dogfish, the steamed samples exhibited no Se_T_ loss, which makes it the safer option to mitigate Hg toxicity. Additionally, the Se/Hg molar ratio data support this observation, where an increase is noticed in the steamed dogfish compared to the raw samples.

## 4. Conclusions

This study addresses the impact of various cooking methods (boiling, grilling, steaming, and frying) on the fate of Hg and Se in three predatory fish species (tuna, swordfish, and dogfish). These culinary treatments affect the Hg_T_ and Se_T_ levels in fish in very distinct ways. The same fish species can behave differently under different cooking methods. Intra- and inter-fish species differences are also observed and must be accounted for when evaluating the impact of cooking on fish. Additionally, there is no association between the behavior of Hg_T_ and Se_T_ due to cooking. 

Two ways of data treatment were carried out to accurately assess the impact of cooking. One dataset presents the composition data in terms of Hg_T_ and Se_T_ in fish at the levels that reach consumers through fish consumption, allowing the assessment of the dietary exposure to these elements if they are consumed in cooked samples (boiling, grilling, steaming, and frying). The other dataset presents the net gains or losses of Hg_T_ and Se_T_ after being submitted to the four performed culinary treatments in comparison to their levels in the raw samples. 

Regarding the food composition levels, in terms of dietary exposure to Hg_T_ and potential Hg_T_ toxicity due to the consumption of this type of cooked fish, the lower risk is encountered for raw swordfish, raw and fried tuna, and steamed dogfish. It is also interesting to note that the highest Se/Hg molar ratio was found in tuna samples (always considerably >1). 

The results related to gain and loss of Hg_T_ and Se_T_ corroborate the previous fish consumption recommendations based on the molar ratios.

Finally, our study demonstrates the difficulty to mitigate Hg toxicity in predatory fish species based on cooking, due to the inconsistency of the effects of cooking even for fish of the same species, most probably due to the difference in the physico-chemical properties of the samples itself, such as fat and water contents, age, etc. 

This study must be followed with optimizing the cooking conditions (using salt and additives, temperature, etc.) in order to ensure a more efficient removal of Hg while preserving fish’s beneficial properties due to nutriments such as total proteins, lipids, and omega acids. Furthermore, this study should be complemented by Hg and Se speciation analysis data, taking into account the varying toxicity of different forms of Hg and the differing counteractive effects of Se species on Hg.

## Figures and Tables

**Table 1:** Optimum operating conditions for the simultaneous determination of total Se and total Hg in fish samples by ICP-MS (Agilent 7700).

Nebuliser	Quartz concentric (Micromist)
Spray chamber	Scott-type double-pass water cooled (2 °C)
RF power	1500 W
Reflected power	<10 W
Plasma gas flow	15.0 L min^−1^
Nebulizer gas flow	~1.0 L min^−1^ (optimised daily)
Auxiliary gas flow	~1.0 L min^−1^ (optimised daily)
Isotopes monitored and detection mode	^202^Hg—conventional (no gaz mode) ^82^Se—use of He as colisison gas

**Table 2:** Se/Hg molar ratio (mean ± standard deviation, *n* = 5) in raw and cooked samples using different culinary treatments, per fish species.

Culinary Treatment	n(Se)/n(Hg) ± SD ^1^ (*n* = 5)		
	Tuna	Swordfish	Dogfish
Raw 1	3.0 ± 1.3	1.1 ± 0.7	0.9 ± 0.6
Boiling	2.4 ± 1.4	0.9 ± 0.8	0.8 ± 0.5
Grilling	2.3 ± 1.6	1.0 ± 0.5	0.8 ± 0.5
Steaming	2.5 ± 1.6	0.9 ± 0.7	1.0 ± 0.6
Raw 2	4.7 ± 5.3	0.6 ± 0.2	1.0 ± 0.3
Frying	4.3 ± 4.2	0.4 ± 0.1	0.9 ± 0.2

^1^ SD = standard deviation.

## Data Availability

Data are contained within the article.

## References

[1] FAO (2020). La Situation Mondiale des Pêches et de L’aquaculture.

[2] Liao W., Wang G., Zhao W., Zhang M., Wu Y., Liu X., Li K. (2019). Change in mercury speciation in seafood after cooking and gastrointestinal digestion. J. Hazard. Mater..

[3] Costa F., Mieiro C., Pereira M., Coelho J. (2022). Mercury bioaccessibility in fish and seafood: Effect of method, cooking and trophic level on consumption risk assessment. Mar. Pollut. Bull..

[4] Mateu M.B., Llovet M., Bonancia B.M., Roig J.L.D., Linares-Vidal V. (2015). Effects of cooking process on the concentrations of mercury, selenium and GPx activity in Tuna (*Thunnus thynnus*). Toxicol. Lett..

[5] Ouédraogo O., Amyot M. (2011). Effects of various cooking methods and food components on bioaccessibility of mercury from fish. Environ. Res..

[6] Cabanero A.I., Madrid Y., Cámara C. (2004). Selenium and mercury bioaccessibility in fish samples. Anal. Chim. Acta.

[7] Bosch A.C., O’Neill B., Sigge G.O., Kerwath S.E., Hoffman L.C. (2015). Heavy metals in marine fish meat and consumer health: A review. J. Sci. Food Agric..

[8] Jitaru P., Adams F. (2004). Toxicity, sources and biogeochemical cycle of mercury. J. Phys. IV.

[9] Kumari S., Amit, Jamwal R., Mishra N., Singh D.K. (2020). Recent developments in environmental mercury bioremediation and its toxicity: A review. Environ. Nanotechnol. Monit. Manag..

[10] Deng L., Li Y., Yan X., Xiao J., Yang R. (2015). Ultrasensitive and highly selective detection of bioaccumulation of methyl-mercury in fish samples via Ag⁰/Hg⁰ amalgamation. Anal Chem..

[11] European Commission (2006). Commission Regulation (EC) No 1881/2006 of 19 December 2006 setting maximum levels for certain contaminants in foodstuffs. Off. J. Eur. Union.

[12] Wood C., Farrell A., Brauner C. (2011). Fish Physiology: Homeostasis and Toxicology of Essential Metals.

[13] Khan M.A., Wang F. (2009). Mercury-selenium compounds and their toxicological significance: Toward a molecular understanding of the mercury-selenium antagonism. Environ. Toxicol. Chem..

[14] Castriotta L., Rosolen V., Biggeri A., Ronfani L., Catelan D., Mariuz M., Bin M., Brumatti L.V., Horvat M., Barbone F. (2020). The role of mercury, selenium and the Se-Hg antagonism on cognitive neurodevelopment: A 40-month follow-up of the Italian mother-child PHIME cohort. Int. J. Hyg. Environ. Health.

[15] Gajdosechova Z., Mester Z., Feldmann J., Krupp E.M. (2018). The role of selenium in mercury toxicity—Current analytical techniques and future. Trends Anal. Chem..

[16] Reilly C. (2006). Introduction. Selenium in Food and Health.

[17] Manceau A., Gaillot A.C., Glatzel P., Cherel Y., Bustamante P. (2021). In Vivo Formation of HgSe Nanoparticles and Hg—Tetraselenolate Complex from Methylmercury in Seabirds—Implications for the Hg-Se Antagonism. Environ. Sci. Technol..

[18] Tinggi U., Perkins A.V. (2022). Selenium Status: Its Interactions with Dietary Mercury Exposure and Implications in Human Health. Nutrients.

[19] Ralston N.V., Ralston C.R., Blackwell J.L., Raymond L.J. (2008). Dietary and tissue selenium in relation to methylmercury toxicity. NeuroToxicology.

[20] Vicente-Zurdo D., Gómez-Gómez B., Pérez-Corona M.T., Madrid Y. (2019). Impact of fish growing conditions and cooking methods on selenium species in swordfish and salmon fillets. J. Food Compos. Anal..

[21] Costa F.D.N., Korn M.G.A., Brito G.B., Ferlin S., Fostier A.H. (2016). Preliminary results of mercury levels in raw and cooked seafood and their public health impact. Food Chem..

[22] Mieiro C., Coelho J., Dolbeth M., Pacheco M., Duarte A., Pardal M., Pereira M. (2016). Fish and mercury: Influence of fish fillet culinary practices on human risk. Food Control.

[23] FAO/INFOODS (2016). Global Food Composition Database for Fish and Shellfish Version 1.0-uFiSh1.0.

[24] Chevallier E., Chekri R., Zinck J., Guérin T., Noel L. (2015). Simultaneous determination of 31 elements in foodstufs by ICP-MS after closed-vessel microwave digestion: Method validation based on the accuracy profile. J. Food Compost. Anal..

[25] Harrington C.F., Merson S.A., Silva T.M.D. (2004). Method to reduce the memory effect of mercury in the analysis of fish tissue using inductively coupled plasma mass spectrometry. Anal. Chim. Acta.

[26] Li Y.-F., Chen C., Li B., Wang Q., Wang J., Gao Y., Zhao Y., Chai Z. (2007). Simultaneous speciation of selenium and mercury in human urine samples from long-term mercury-exposed populations with supplementation of selenium-enriched yeast by HPLC-ICP-MS. J. Anal. At. Spectrom..

[27] FDA (2023). Mercury in Food and Dietary Supplements. https://www.fda.gov/food/environmental-contaminants-food/mercury-food-and-dietary-supplements.

[28] ANSES (2023). Ciqual French Food Composition Table. https://ciqual.anses.fr/.

[29] EFSA NDA Panel (2022). Scientific opinion on the tolerable upper intake level for selenium. EFSA J..

[30] Burger J., Gochfeld M. (2012). Selenium and mercury molar ratios in saltwater fish from New Jersey: Individual and species variability complicate use in human health fish consumption advisories. Environ. Res..

[31] Braaten H.F.V., de Wit H.A., Harman C., Hageström U., Larssen T. (2014). Effects of sample preservation and storage on mercury speciation in natural stream water. Int. J. Environ. Anal. Chem..

[32] Salim R. (1987). Effect of storage on the distribution of trace elements (lead,’ cadmium, copper, zinc and mercury) in natural water. J. Environ. Sci. Health Part A Environ. Sci. Eng..

[33] Selin N.E. (2009). Global Biogeochemical Cycling of Mercury: A Review. Annu. Rev. Environ. Resour..

[34] Burger J., Gochfeld M. (2011). Mercury and selenium levels in 19 species of saltwater fish from New Jersey as a function of species, size, and season. Sci. Total. Environ..

[35] Schartup A.T., Thackray C.P., Qureshi A., Dassuncao C., Gillespie K., Hanke A., Sunderland E.M. (2018). Climate change and overfishing increase neurotoxicant in marine predators. Nature.

[36] Milea Ș.-A., Lazăr N.-N., Simionov I.-A., Petrea Ș.-M., Călmuc M., Călmuc V., Georgescu P.-L., Iticescu C. (2023). Effects of cooking methods and co-ingested foods on mercury bioaccessibility in pontic shad (*Alosa immaculata*). Curr. Res. Food Sci..

[37] Perugini M., Zezza D., Tulini S.M.R., Abete M.C., Monaco G., Conte A., Olivieri V., Amorena M. (2015). Effect of cooking on total mercury content in Norway lobster and European hake and public health impact. Mar. Pollut. Bull..

[38] Martin D., Lobo F., Lavison-Bompard G., Guérin T., Parinet J. (2020). Effect of home cooking processes on chlordecone content in beef and investigation of its by-products and metabolites by HPLC-HRMS/MS. Environ. Int..

